# Powder-Based 3D Printing for the Fabrication of Device with Micro and Mesoscale Features

**DOI:** 10.3390/mi11070658

**Published:** 2020-06-30

**Authors:** Seow Yong Chin, Vishwesh Dikshit, Balasankar Meera Priyadarshini, Yi Zhang

**Affiliations:** 1 HP-NTU Digital Manufacturing Corporate Lab, Nanyang Technological University, 50 Nanyang Ave, Singapore 639798, Singapore; yongchin.seow@ntu.edu.sg (S.Y.C.); vishdixit@ntu.edu.sg (V.D.); balasankar.mp@ntu.edu.sg (B.M.P.); 2 School of Mechanical and Aerospace Engineering, Nanyang Technological University, 50 Nanyang Ave, Singapore 639798, Singapore

**Keywords:** 3D printing, 3D-printed devices, powder bed fusion technologies, micro and mesoscale 3D printing, minimum feature size, 3D-printed scaffold

## Abstract

Customized manufacturing of a miniaturized device with micro and mesoscale features is a key requirement of mechanical, electrical, electronic and medical devices. Powder-based 3D-printing processes offer a strong candidate for micromanufacturing due to the wide range of materials, fast production and high accuracy. This study presents a comprehensive review of the powder-based three-dimensional (3D)-printing processes and how these processes impact the creation of devices with micro and mesoscale features. This review also focuses on applications of devices with micro and mesoscale size features that are created by powder-based 3D-printing technology.

## 1. Introduction

Additive manufacturing, also termed three-dimensional (3D) printing, is a process that transforms the computer aided-design model into a true 3D object using various materials. The 3D printing provides unparalleled flexibility that enables layer-by-layer construction of functional parts with complex shapes and geometries. The 3D printing emerges as a viable alternative to conventional industrial production technology [1,2,3]. Much effort has been made to characterize the durability, surface finishing and mechanical properties of 3D-printed objects [4,5,6,7,8,9,10,11,12]. Nevertheless, concerns are raised for applications that subject 3D-printed parts to repeated stress that may cause fatigue failure [12,13,14,15,16]. The 3D-printing revolution has been seen by many as one of the technologies that will form the industrial revolution 4.0. Compared to the conventional subtractive manufacturing methods, 3D printing enables high design complexity and shorter design cycle [17,18,19].

3D printing is primarily classified into seven categories: (1) binder jetting, (2) powder bed fusion (PBF), (3) directed energy deposition, (4) material jetting, (5) vat polymerization, (6) material extrusion and (7) sheet lamination [20,21]. The 3D printing could also be categorized based on the primer materials into the categories of liquid-, solid- and powder-based processes [22]. The powder-based process is one of the most significant and popular class of 3D-printing techniques [23,24,25,26,27,28]. This popularity is due to the high reusability rate of the powder material, faster production speed, strong functional parts, lower cost, no or minimum support structures, different fields of application and a large range of compatible materials [2,3,12,23,24,25,26,27,29,30,31,32,33,34,35,36,37,38,39,40]. The burgeoning field of 3D printing has changed the way products are manufactured in many industries by offering a higher degree of freedom in design and fabrication with a wide range of materials [41,42,43,44,45].

Several industrial sectors including biomedical, industrial, chemical, aerospace, electronics, communications and energy, have the need to miniaturize their products for various purposes. The conventional micromachining techniques are unable to achieve true 3D structures and face challenges in manufacturing complex shapes [46,47]. Significant effort has been put into developing micro 3D printing processes based on stereolithography (SLA), material/binder jetting, micro selective laser melting/sintering processes and micro cladding [48,49,50,51,52,53,54,55,56,57,58,59,60]. Although micro 3D printing can print true 3D microfeatures, its throughput is too low for industrial scale manufacturing [48,49,50,51,52,53] and most of these techniques are still under development phase. In comparison to other 3D-printing processes, industrial powder-based 3D-printing processes have significantly higher throughput, but limited resolution [17,50,52,61,62]. To achieve both high-resolution and high-throughput manufacturing, a multiscale and multi-print speed 3D-printing process is desired to print microscale features with a high-resolution and the rest of the part at a high speed. Powder-based 3D printing is well-suited for industrial scale manufacturing due to its high throughout, high scalability, post-printing processability and wide material selection [7,24,29,48,63,64,65,66,67]. However, industrial powder-based 3D-printers are often limited by the lack of transparent material and relatively low printer resolution and accuracy [17,41,44,62,68]. Based on the smallest feature of a 3D-printed component, we categorize powder-based 3D printing into nanoscale (<100 nm), microscale (100 nm to 100 µm), mesoscale (100 µm to one millimeter) and macroscale (>one millimeter). In this review, we will examine the capability of various powder-based 3D-printing processes in resolving micro and mesoscale features with a size ranging from 10 µm to 1 mm in size (with several examples slightly larger than one millimeter) and evaluate their suitability for various applications. At present, it is impractical to fabricate nanoscale features with powder-based 3D-printing processes [48,50,69,70], and the advances in macroscale powder-based 3D printing have already been extensively reviewed [4,12,27,28,37,39,63,64,65,68,71,72,73,74,75,76,77,78,79]. Therefore, these two areas are beyond the scope of this review. Although the 3D-printed components discussed in this review may have a large overall size, they are categorized according to the size of their smallest feature.

## 2. Powder-Based 3D-Printing Modalities and Their Resolution

**Figure 1:**
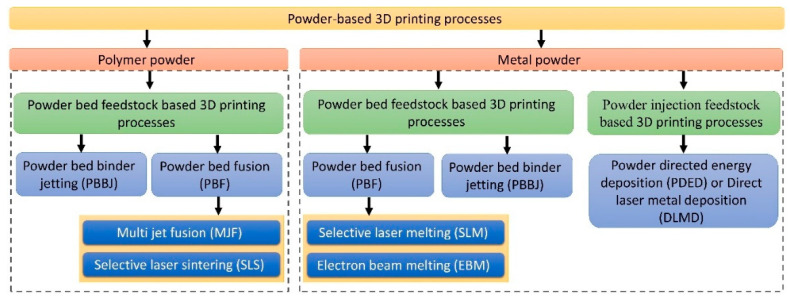
Classification of powder-based three-dimensional (3D) printing.

Powder-based 3D-printing processes are very common in polymer 3D printing as well as in metal 3D printing [23,24,26]. Figure 1 shows the schematic diagram for the classification of powder-based 3D-printing processes. Out of the seven 3D-printing categories, powder-based 3D-printing processes cover only three categories; binder jetting, PBF and directed energy deposition. Both powder injection (or blown powder) and powder bed feedstock mechanisms are used for metal powder-based 3D-printing process. Till now, only powder bed feedstock mechanism is used for polymer powder-based 3D-printing process. Powder injection-based 3D printing, such as powder directed energy deposition (PDED), utilizes an energy source to melt the blown powder deposited by a nozzle. In this scenario, the PDED process is typically used with metal powders only. PDED is popular for part repair or modification [80]. However, PDED is also capable of manufacturing metal alloy-based near net-shape parts [81].

As shown in Figure 1, powder bed 3D-printing processes can be subdivided into a powder bed binder jetting (PBBJ) and PBF. PBBJ utilizes a liquid binding agent to glue the powders in selected regions of a powder bed. Generally, post-printing sintering is required to improve mechanical properties of the PBBJ-printed objects [63,66,78,82,83,84]. On the other hand, PBF technology, first introduced by Deckard and Beaman [85], utilizes energy source (thermal, laser, electron beam or infrared source) to melt or sinter powders in selected regions on the powder bed [23,86]. Various kinds of materials, such as sand, calcium carbonate, polymer powder, metal powder, ceramic powder and composite powder, can be used in the powder bed 3D-printing process. Support structures are generally not required in the powder bed 3D-printing process as the un-fused or un-glued powders function as the supporting structure. After the object is created, the un-fused or un-glued powder is removed and, in some cases, reused. Various techniques are used to remove the un-fused powder such as mechanical brushing, ultrasonication, shot-peening, compressed air and bead blasting [23,27,82,86]. The minimum feature size recommended as per standard manufacturing guidelines for the polymer PBF process and metal PBBJ is around 0.5 mm and 1–2 mm, respectively [87].

**Figure 2:**
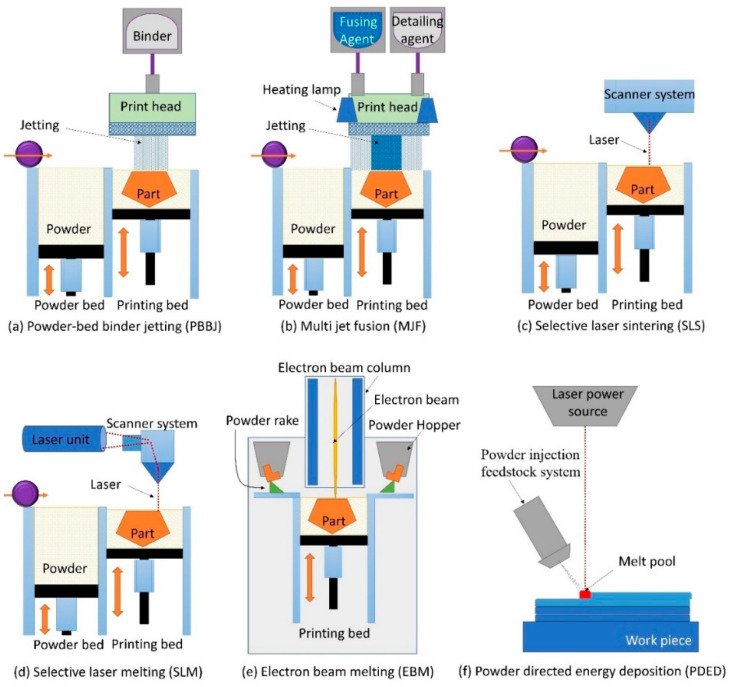
Schematic diagram of the various powder-based 3D-printing processes. (**a**) Powder bed binder jetting (PBBJ): jetting of chemical binder; (**b**) multi jet fusion (MJF): jetting of fusing and detailing agent. Infrared heat absorbing polymer fusion method; (**c**) selective laser sintering (SLS): laser powered sintering method; (**d**) selective laser melting (SLM): laser powered localized material melting method, (**e**) electron beam melting (EBM): electron beam powered localized metal melting method; (**f**) powder directed energy deposition (PDED) or direct laser metal deposition (DLMD): laser powered injected-material melting method.

Figure 2 shows the basic working principle of main established industrial powder-based 3D-printing processes. The main powder-based 3D-printing processes are explained as follows:

### 2.1. Powder Bed Binder Jetting

**Figure 3:**
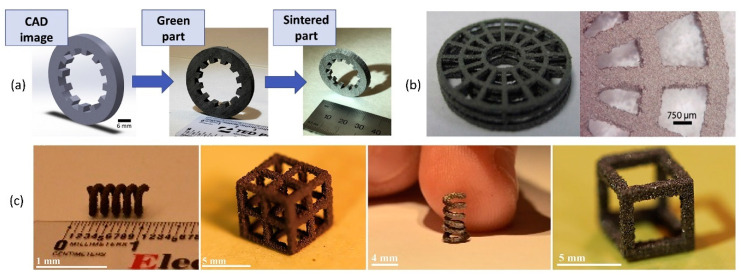
PBBJ 3D-printed parts. (**a**) Computer-aided design image to sintered part. Reproduced with permission from [92]; (**b**) PBBJ 3D-printed mesh structures with powder size < 20 µm. The designed width is 400 µm, and the measured width ranges from 650–770 µm. Reproduced with permission from [100]; (**c**) example of PBBJ 3D-printed components with complex shapes. Reproduced with permission from [101].

Early work on PBBJ came from a research group at Massachusetts Institute of Technology [88] (Figure 2a). The PBBJ process makes use of liquid binding agent to join adjacent powder particles in each layer, after which the printing bed is lowered to allow the deposition of another powder layer as shown in Figure 2a and Figure 3a. This method can 3D-print a green part with a range of different materials, including polymer, metals, sand, ceramics, chalk powder, mixed materials and composites [61,63,76,83,89,90,91]. However, the post-processing (e.g., debinding) of green parts could be time-consuming, and the green parts often are not suitable for designed applications. Generally, sintering is required to reduce porosity and improve mechanical preformation of the 3D-printed green part, and infiltration is sometimes required (Figure 3a) [82,91,92,93,94,95,96]. After fabrication, hot isostatic pressing could be used to increase the density of the green parts [63,66,82,83,93]. PBBJ allows the printing of large objects. Typical feature size for ceramic PBBJ is reported around 22–500 µm [97,98], and the typical feature size of metal and polymer PBBJ is 100 µm [99] depending on the size of the powder used for printing [98]. This 3D-printing process is ideal for the production of porous ceramic components [71]. The strength and the surface roughness of the green part is directly dependent on the powder size and binder spreading. The 3D-printed mesh structure (Figure 3b) using finer powders (<20 µm) shows a better surface roughness and higher green strength. However, it is also reported that the over spreading of the binder may reduce dimensional accuracy of 3D-printed parts. As shown in Figure 3b, the printed dimension appears bigger than the designed dimension (650–770 µm vs. 400 µm) as a result of binder over spreading [100]. Figure 3c shows that components 3D-printed by PBBJ using Ni–Mn–Ga magnetic shape-memory alloys powder [101] have complex shapes and different porosities.

### 2.2. Multi Jet Fusion

**Figure 4:**
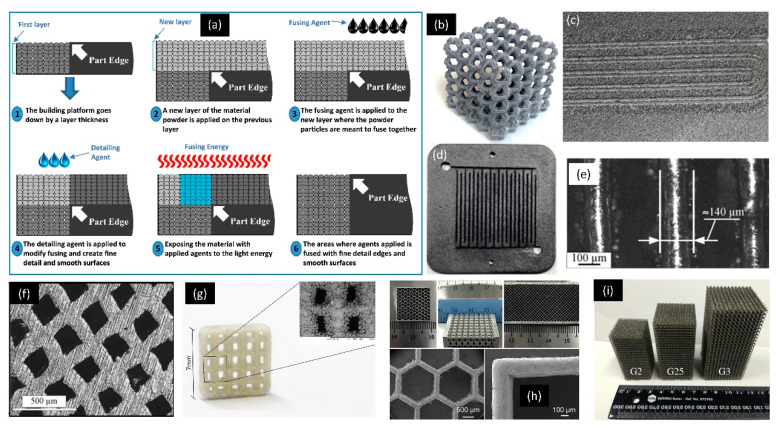
Concept of MJF 3D printing and example of PBF 3D-printed parts. (**a**) Schematic presentation of MJF 3D-printing process. Reproduced with permission from [106]; (**b**) MJF 4200 3D-printed lattice structure, HP PA12 material. Reproduced with permission from [106]; (**c**) MJF 4200 3D-printed microchannel, HP PA12 material, channel size: 300-µm width, 250-µm depth, channel; (**d**) typical flow field design 3D-printed by SLS with channel size, width of 1.5 mm, depth of 1.5 mm and the land width of 1.0 mm. Reproduced with permission from [121]; (**e**) fine-walled 3D-printed by SLM 3D-printing process, wall thickness 140 μm. Reproduced with permission from [122]; (**f**) micro square channels 3D-printed by SLM 3D-printing process, channel size is 150 μm × 150 μm, the wall thickness is 120 μm. Reproduced with permission from [122]; (**g**) thin lattice structure 3D-printed by SLM 3D-printing process of glass. Reproduced with permission from [123]; (**h**) microfeatures manufactured by micro-SLM 3D-printing process. Reproduced with permission from [48]; (**i**) EBM 3D-printed Ti–6Al–4V gyroid scaffolds. Reproduced with permission from [124].

HP, Inc; (HP) introduced the multi jet fusion (MJF) 3D-printing process, a special type of PBF process [20,102,103]. This technique is like PBBJ and an inkjet system in terms of the material feedstock [66]. However, the fusing mechanism of MJF is entirely different from PBBJ and SLS because it fuses the material with the help of infrared heating and a chemical fusing agent [67,103,104]. Figure 4 provides a schematic diagram of MJF 3D-printing process and various examples of PBF 3D-printed parts. Figure 2b and Figure 4a show the detailed working principle of MJF 3D-printing process. In this process, a thin layer of powder is first spread from the powder bed feedstock to the print bed that is pre-heated to a uniform temperature. Then, as per print requirement, the fusing agent and detailing agent are dispensed onto selected regions of the powder bed using HP thermal inkjet system (print head). After this, the print bed is exposed to an infrared source that allows the final fusing of the powder in selected regions (Figure 4a). The fusing agent is basically a radiation absorbing material infused into the powder bed [23,32,103,105]. Hence, the regions on the powder bed with infused fusing agent absorb more heat, resulting in melting and fusing the polymer powders. The detailing agent (non-absorbing material) is dispensed along the fused boundary to counteract the effect of the fusing agent. It cools down the temperature of the powder bed. Detailing agent also reduces the thermal distortion in 3D-printed parts and improves the accuracy and detail of prints close to the fused boundary [106,107]. The extended melt and fusing time results in better fusing. The current powder materials available for MJF are polyamide (PA) 11, PA 12 and thermoplastic polyurethane (TPU). This emerging 3D-printing modality is suitable for low volume production of parts with exceptional mechanical strength, high reusability rate of the powder material, faster production speed as an alternative to injection molding [23,67,103,104]. MJF is capable of printing functional mechanical parts/devices, biomedical lattices structures, medical orthotics and prosthetics, mechanical tools and fluid-tight devices [34,67,103,106,108,109,110,111]. Figure 4b shows exemplary high-performance lattice structures 3D-printed by MJF 4200 with cell units ranging from 1.134 to 2.246 mm [106]. The recommended minimum feature size with standard print quality is around 0.5 mm [87]. However, the typical feature size can be further optimized down to 250 µm (Figure 4c). Figure 4c shows MJF 3D-printed microchannel of 300-µm width and 250-µm depth.

High speed sintering (HSS) is another similar 3D-printing process like MJF. The HSS 3D-printing process also jets an infrared absorbing ink that polymerizes the powder under infrared heat [112,113,114,115,116,117,118,119]. However, HSS 3D-printing process does not use detailing agent or transforming agent to improve accuracy, print detail, color and change material property [107,120].

### 2.3. Selective Laser Sintering

Selective laser sintering (SLS) is the most common polymer PBF process. This technique utilizes high-energy laser beam to scan over a layer of powder as shown in Figure 2c. Layers of sintered material are fused together, forming complex 3D structures. The basic repetitive building sequence is (a) lowering of the printing bed equivalent to layer thickness, (b) deposition of powder on the printing bed and (c) scanning of a laser beam selectively on the printing bed [39]. The printing bed is pre-heated to sufficient temperature with the filling of inert gas to form a nonoxidative chamber. The building material can be polymer, glass, ceramic and polymer composite [23]. As reported, SLS 3D-printing process can produce a typical feature size around 200 μm [125]. SLS 3D-printing process can produce typical feature size around 40–100 μm [50,58,126]. The SLS 3D-printing process is suitable for processing a range of materials, such as nylon, polycarbonate, composite of nylon-glass, ceramics, polymer-metal powders, hydroxyapatite, etc. [127]. Figure 4d shows an example of SLS 3D-printed conventional flow field design of bipolar plates with minimum feature size of 1 mm [121]. A similar process is also followed in metal 3D printing, which is generally categorized as direct metal laser sintering (DMLS), direct selective laser sintering and laserCUSING [39,128]. Using standard settings, DMLS can produce a minimum feature size of around 500 μm. However, the minimum feature size reported in other studies has reached 380 μm and 153 μm, respectively, for standard DMLS and high-resolution DMLS [52].

### 2.4. Selective Laser Melting

Selective laser melting (SLM) 3D-printing process and SLS 3D-printing process look very similar, as both 3D-printing processes use powder bed feedstock and laser energy source. However, in SLM powder particles are fully melted during the fusing process due to significantly higher laser powder [80,129]. This process is also commonly mentioned as direct metal laser melting or laser powder bed fusion (LPBF). Figure 2d shows a representation of the basic working principle of the SLM 3D-printing process. This process is more suitable for creating dense metal parts. The surface roughness of the specimen made by SLM is higher than that of the specimen made by SLS. The bonding strength of SLM 3D-printed part is higher than that of SLS 3D-printed part [27,65,130]. Generally, commercial SLM 3D-printing process employs 20–50 µm metal powder particles to print 20–100 µm thick metal layers [48]. It is difficult to further reduce the size of metal particles due to safety concerns and technical challenges such as poor spreadability [131,132]. The minimum feature size reported for SLM is in the range of 40–200 µm [29,133]. Figure 4e,f shows an example of SLM 3D-printed stainless steel microchannels with a square cross-section and a wall thickness of 120 μm [122]. It is also possible to print glass-like materials using SLM, which could achieve a minimum channel size of 1.1 mm (Figure 4g) [123]. Recently, micro-SLM 3D-printing process has been developed [48]. Micro-SLM 3D printing is capable of printing microscale features as small as 60 μm with a minimum surface roughness (Ra) of 1.3 μm (Figure 4h) [48].

### 2.5. Electron Beam Melting

The 3D-printing process of electron beam melting (EBM) employs an electron beam as the heat source to fully melt the powder in a vacuum environment as depicted in Figure 2e. EBM 3D printing can only be used to construct metal 3D parts. The metal powder is pre-heated before being fused by an electron beam. EBM does not require support structures during printing. This technique is often utilized to fabricate biomedical implants and aeronautic parts made of materials such as titanium alloy [134]. The spot size of the electron beam is slightly wider than the laser beam [135]. The EBM 3D-printing process can produce a typical feature size around 100–200 μm [33,136]. EBM 3D-printing process is a viable substitute for SLM 3D-printing process. Because EBM 3D-printing process uses a high-density energy source, it is possible to produce void-free components. Figure 4i shows three sets of EBM 3D-printed gyroid scaffolds consisting of various unit cells with a minimum feature size of 0.5 mm [124].

### 2.6. Powder Directed Energy Deposition

**Figure 5:**
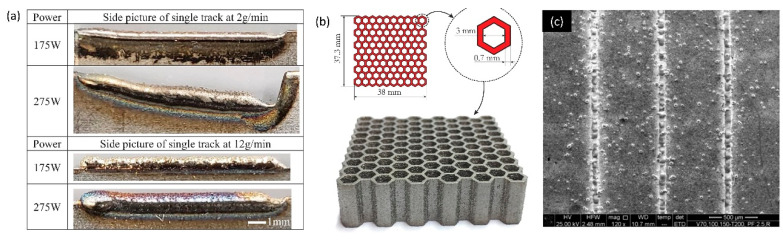
Example of PDED/DLMD 3D-printed parts. (**a**) Single track material deposition by PDED 3D-printing process. Reproduced with permission from [139]; (**b**) laser engineered net shaping (LENS) 3D-printed cellular honeycomb structures. Reproduced with permission from [140]; (**c**) NiTi single tracks printed by PDED 3D-printing process. Reproduced with permission from [144].

PDED system consists of a robotic arm (4–5 axis), a powder injection feedstock and a focused laser as the heat source. As the laser is mainly used as the thermal energy source in this technology, PDED is also known as direct laser metal deposition (DLMD) [81]. However, electron beam, plasma and electrical arc are also alternative thermal energy source used in this technology [137]. Figure 2f shows the working principle of PDED/DLMD process. Lateral or off-axis, continuous coaxial and discontinuous coaxial powder injection are the three possible powder injection feedstock mechanisms used in the PDED/DLMD 3D-printing process [80]. The injected powders are immediately melted by the heat source when they enter the focused heating zone, and the fused material is deposited on top of the targeted surface. The deposited material gradually solidifies and metallurgically bonded with the parent substrate material [64,81]. The robotic arm empowers large-space printing, printing on curved or complex surface and repairing/cladding of existing parts [64]. According to the powder injection feedstock process, heat source and motion-control method, different PDED/DLMD systems have been developed such as laser engineered net shaping (LENS), laser metal deposition, direct laser deposition, direct light fabrication, laser deposition welding and powder fusion welding (PFW) [64,81]. The minimum feature sizes are reported between 500 μm to 3 mm for PDED/DLMD powder-fed process [52]. PDED/DLMD is a highly flexible 3D-printing process which can be applied to various applications for medical device manufacturing to medium and large-scale repair [138]. The thickness of PDED/DLMD 3D-printed layer can be controlled by tuning the laser power and mass flow rate of the blown powder. The minimum feature achieved by PDED/DLMD is 0.5 mm (Figure 5a) [139]. Figure 5b shows an example of LENS 3D-printed cellular honeycomb structures with a wall thickness of 0.7 mm [140]. The micro-PDED/DLMD process (μ-PDED/DLMD) has achieved minimum feature size of 0.5 mm [141,142,143,144]. Figure 5c shows a NiTi single track printed by μ-PDED/DLMD with a width 20 μm.

Table 1 summarizes the applications and process capabilities of different powder-based 3D-printing processes. The applications of these 3D-printed micro and mesoscale features are discussed in Section 3 in detail.

## 3. Powder-Based 3D Printing for Fabricating Devices with Micro and Mesoscale Features

Powder-based 3D-printing covers a broad range of applications in industry and academia [35]. In this section, devices that are fabricated by powder-based 3D printing and have micro and mesoscale features that are crucial for intended applications will be discussed in detail. All devices will be compared and summarized in Table 2 in terms of the powder-based 3D-printing process, smallest feature size and material.

### 3.1. Biomedical, Chemical, and Pharmaceutical Applications

Powder-based 3D printing demonstrates important applications in biomedical, chemical and pharmaceutical devices. In, general, powder-based 3D-printing process are capable of fabricating device with minimum feature sizes around 50 µm (Table 1) [29,33,99,133,136]. Powder-based 3D printing allows the manufacturing of various complex non-porous and porous structures. In the context of biomedical applications, 3D-printed lattice structures help the bone ingrowth that eventually leads to improved implant durability. Manufacturing of medical implants are the one area that benefits most from powder-based 3D-printing technology [27,152,157,158,159,160,161]. The 3D printing medical implants enables customized implant design and modification, allows fast design iteration, reduces the cost for low-quantify production and leads to better clinical outcomes. To date, applications of powder-based 3D printing in the biomedical field is still limited to parts with meso and macroscale feature size, such as customized microwell plates to house membranes of different sizes [162]. The 3D-printed devices fabricated by powder-based 3D-printing processes have been applied to biomedical, chemical, healthcare industry [163,164,165,166].

**Figure 6:**
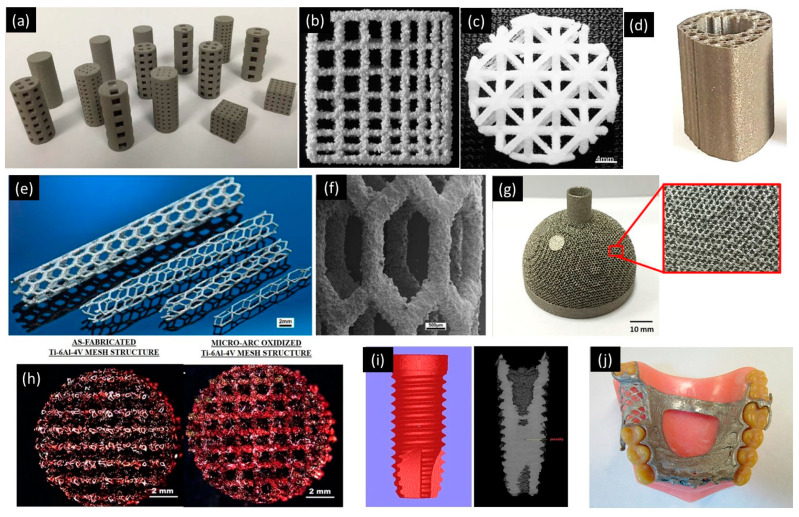
Examples of various powder-based 3D-printed scaffolds. (**a**) PBBJ 3D-printed specimens of various sizes, shapes and lattice structure designs. Reproduced with permission from [171]; (**b**) PBBJ 3D-printed grid structure with wall thickness of 330 μm. Reproduced with permission from [161]; (**c**) SLS 3D-printed polycaprolactone scaffold with pore size 40–400 μm. Reproduced with permission from [126]; (**d**) Ti–6Al–4V implant prototypes manufactured by direct metal laser sintering (DMLS). Reproduced with permission from [177]; (**e**) cardiovascular stents 3D-printed by SLM 3D-printing process. Reproduced with permission from [178]; (**f**) scanning electron microscope (SEM) image of cardiovascular stents. Reproduced with permission from [178]; (**g**) EBM 3D-printed porous acetabular cup implant. Reproduced with permission from [27]; (**h**) EBM 3D-printed mesh structures for intercellular cell communication and osteoincorporation. Reproduced with permission from [179]; (**i**) microcomputed tomography image of the Ti–6Al–4V dental implant 3D-printed by EBM 3D-printing process. Reproduced with permission from [180]; (**j**) sintered partial denture framework 3D-printed by PBBJ 3D-printing process. Reproduced with permission from [181].

Powder-based 3D-printing processes can create various types of pore architecture with microporosity (40–100 μm) or macroporosity (>100 μm). Pore architecture and pore size are the critical parameters for cell migration into the scaffolds [126,144,152,167,168,169,170]. Generally, the minimum designed pore size is the minimum feature size of the 3D-printed scaffolds, whereas the naturally occurring micropores as a result of the material properties are not considered the minimal feature of the 3D-printing process. Figure 6 shows some examples of the powder-based 3D-printed scaffolds. Figure 6a,b shows the PBBJ-printed scaffolds with a minimum feature size ranging from 330 μm to 1 mm [161,171]. Polycaprolactone scaffold manufactured by SLS 3D-printing process was reported with minimum micropores size 40–400 μm (Figure 6c), which was found effective for cell attachments [126,172]. Pores can be designed with a size down to 0.5–1.2 μm for ceramic-based scaffold, and it has been 3D-printed by SLS 3D-printing process successfully [173,174].

SLM 3D printing is also a good candidate for the fabrication of customized bionic implant with Young’s modulus and yield strength comparable to human bones to facilitate bone tissue regeneration [175]. The minimum feature size of SLM 3D-printed bionic implant was approximately 0.26 mm. The pore size of the hexagonal mesh within the implant was customizable between 60 µm and 265 µm. Structural implants with multilayer hexagonal mesh structure were important to reduce stress shielding and enable bodily fluid to transport through the implant to promote tissue regeneration. Another similar example of SLM 3D-printed porous implants for the lower jaw restoration was demonstrated to have a minimum feature size of approximately 1 mm. By varying the porosity, the 3D-printed implant was able to sustain compressive pressures ranging from 20–350 MPa [176]. Figure 6d shows an example of bone implant 3D-printed with porous Ti–6Al–4V using DMLS 3D-printing process. The average pore size of the implant is 723 μm [177]. Complex Zn implant structures with a strut diameter of 200 μm was successful demonstrated using SLM 3D-printing process [178] (Figure 6e,f).

Customized implants with mesoscale features of approximately 0.3 mm have been fabricated using EBM 3D-printing process. Such structures could not be achieved using conventional machining or molding techniques. Each implant is customized by modeling with individual’s anatomic data obtained from medical imaging (Figure 6g) [27]. In the field of bioreactors, EBM 3D printing helped to create Ti–6Al–4V discs with a thickness of 2 mm for mouse fibroblast cell culture [182]. The orientation of the part during 3D printing was tested to investigate how it would affect the proliferation and the attachment of cells. The results showed that the chamber printed at 90° and 55° orientation enabled the cells to spread to a wider area compared to the horizontal orientation. An EBM 3D-printed mesh structure was used to investigate cell-to-cell communication, including proliferation, synthesis of extracellular and intracellular proteins and mineralization (Figure 6h) [179]. The minimum feature size of the mesh was approximately 0.5 mm. A layer of bioactive titania with a pore size of 1–3 µm was applied on the surface of the 3D-printed complex porous structures. This structure would facilitate oxygen and nutrient transfer to the cells. Plasma electrolytic oxidation applied on the surface was an important step to improve the bioactivity of the mesh structures. A similar anodized 3D-printed Ti–6Al–4V mesh structure fabricated by EBM 3D printing was used for mouse pre-osteoblast cell culture [183]. The surface of the alloy was modified to incorporate titania nanotubes with an average pore size of 80 nm to promote the expression of proteins. The bioactive oxide layer with nanoscale pores residing within the interconnected 3D-printed mesh created a natural bone-like environment for bone formation. EBM 3D printing was also used to form interconnected foamed structure with ligaments for cell culture [184]. The foamed structure was favorable for cell migration, communication and mineralization of osteoblasts. The minimum average pore size within the foam structure was 0.7 mm. A step further to use EBM 3D-printed implants in preliminary human clinical trial was demonstrated successfully [185]. Different porosity within the mesh and foam structures with variable stiffness and elastic modulus were designed into the 3D-printed implant to match both soft and hard bones. By seeding osteoblast and endothelial cell in Matrigel embedded in the implant and inducing hypoxia, the entire 3D-printed porous structure was fully colonized by cells. Figure 6i shows an example of EBM 3D-printed rough and porous Ti–6Al–4V dental implants with mesoscale features (thread width) around 500 µm [180]. The rough and porous surface of the Ti–6Al–4V dental implants facilities bone ingrowth and strengthens bone bonding. PBBJ 3D-printing process is also capable of manufacturing patient-specific dental implants. Patient-specific complex metal partial denture framework (Figure 6j) was fabricated by PBBJ 3D-printing process. In these dental applications, it was found that a wall thickness of 0.5 mm is a practical lower limit [181]. Patient-specific ankle-foot orthoses (AFO) for stroke patients was fabricated using MJF 3D-printing process. The 3D-printed AFO significantly improved the speed and stride length of the stroke patients. The minimum feature (thickness) size of the PA12 AFO was around 1.2 mm [108].

**Figure 7:**
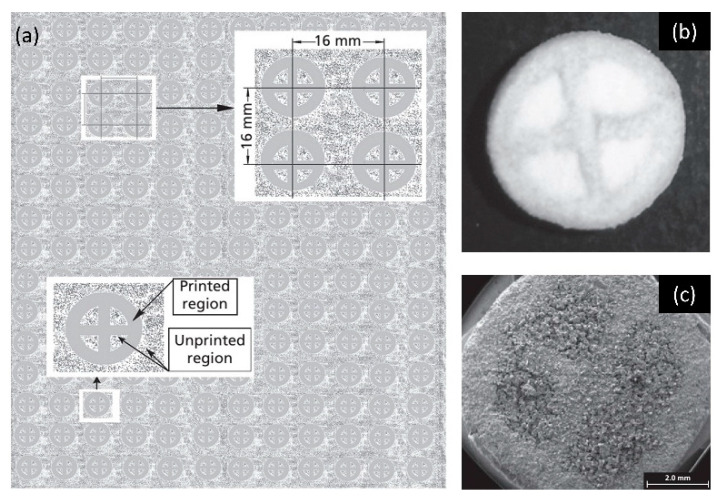
Example of fast-disintegrating drug delivery devices 3D-printed by PBBJ 3D-printing process. (**a**) computer-aided design model of fast-disintegrating drug delivery device. Reproduced with permission from [186]; (**b**,**c**) SEM image of PBBJ 3D-printed fast-disintegrating drug delivery device. Reproduced with permission from [186].

Drug delivery systems have also been fabricated by powder-based 3D printing [186,187,188,189,190]. A drug delivery system with internal compartments was fabricated using a customized binder jet 3D-printing process (Figure 7a–c) [186]. The exterior of the drug delivery system was a cylindrical tablet made of fused powder. Walls made of fused powder partition the interior of tablet into several compartments that are filled with loose drug powder. The smallest feature in the lateral dimension of tablet was estimated to be around 1 mm. This 3D-printed drug tablet showed acceptable pharmaceutical properties with an average disintegration time of about 23 s and an average wetting time of 68 s. It showed a rapid burst release and delivered more than 98% of its drug load in 2 min. This is an important area of research where powder-based 3D-printing process could play a major role. Powder bed 3D-printing process enables the fabrication of drug delivery systems with arbitrary composition, geometries and shapes that could be tuned to control the drug release profile [187,188,189,190]. An interesting cell encapsulation device was fabricated using SLS 3D printing [191]. A patterned macrocapsule with smallest feature ranging from 0.5 mm to 1 mm was 3D-printed by SLS to encapsulate cell-containing microcapsules for cell-based therapy. The porosity, which was controlled by the sintering conditions, was used to adjust the oxygen and nutrient exchange as well as the vascularization process. The 3D printing also helped to achieve rapid prototyping of different biocompatible materials. Similarly, SLS 3D printing can also be used to create an orally disintegrating tablet with a thickness of 2 mm [192]. Different drug release profile can be achieved by varying the SLS 3D-printing parameters. The tablet only needs 4 s to fully dissolve in water due to the reduced density and increased porosity

**Figure 8:**
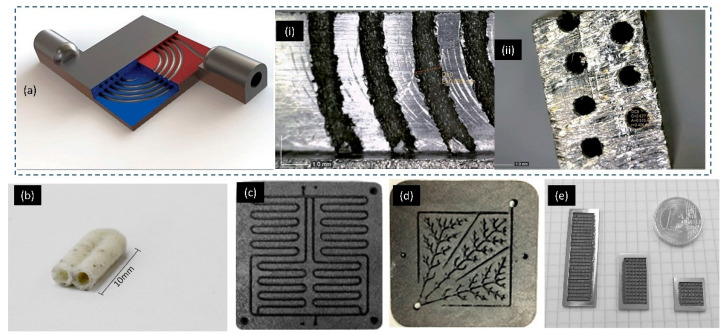
Example of various powder-based 3D-printed fluidic applications. (**a**) SLM 3D-printed capillary liquid chromatography, (0.9 mm inner diameter. × 60 cm length). Reproduced with permission from [195]; (**b**) thin glass tubing 3D-printed by SLM 3D-printing process. Reproduced with permission from [123]; (**c**) SLS 3D-printed graphite composite-based bipolar plates, channel width—1.5 mm and depth—1.5 mm. Reproduced with permission from [197]; (**d**) bio-inspired flow field designs 3D-printed by SLS 3D-prining process, land width of 1.0 mm. Reproduced with permission from [121]; (**e**) stainless steel flow field plates 3D-printed by SLM 3D-printing process, channel size 500 μm. Reproduced with permission from [196].

SLS 3D printing was employed to print graphite composite with mesoscale features down to 1 mm for fuel cell application [193]. The ability of 3D printing to create sophisticated 3D architectures allowed further improvement on the performance of fuel cells. The sophisticated 3D structures within the fuel cell, including channels with multiple outlets or inlets, micro-ridges and flow-through electrodes, accelerated the mass transfer in the depletion layers. The fuel cell improved its performance by implementing these designs to tackle reactant depletion and crossover issues. Powder bed 3D printing showed great promises for the fabrication of the next-generation fuel cells by offering on-demand and flexible manufacturing. SLM 3D printing was used to advance the development of reverse-phase liquid chromatography [194,195]. A titanium (Ti–6Al–4V) alloys complex chromatographic column with an internal monolithic phase was created using SLM 3D printing (Figure 8a). The minimum feature size of the complex internal channel was 0.9 mm [195]. The channel was filled with porous polymer monolith to separate intact proteins and peptides from mixtures. In addition, SLM 3D-printing process was capable of producing glass (soda lime silica)-based closed mesofluidic channels with minimum internal diameter of 1.1 mm (Figure 8b). These highly porous glass-based 3D-printed structures could be used as scaffolds or catalysts structured (Figure 4g and Figure 8b) [123]. Figure 4d and Figure 8c–e show a few examples of 3D-printed flow field plates for micro fuel cell [121,196,197]. The minimum feature (channel) size of the graphite composite-based bipolar plates fabricated by SLS 3D-printing process is down to 1 mm [121,197]. In comparison, the minimum feature (channel) size of stainless steel flow field plates goes down to 500 μm [196].

SLS 3D-printing process was used to fabricate filters using porous material with a pore size of approximately 20 µm. The filter thickness is 1.5 mm. Materials with high porosity, such as metal–organic complex copper (II) benzene-1,3,5-tricarboxylate, was used as the primer powder for SLS 3D printing. The X-ray analysis pointed out that the inherent structure of metal–organic-framework (MOF) is intact. When using PA 12 as the supporting matrix to hold the MOF materials, the 3D-printed structure could be used as filters catered for various applications [198]. SLS 3D-printing technology also helped to create filters that could withstand harsh environment with high pressure, temperature, corrosive wear and tribomechanics [199]. The filter could remove harmful gases during crude oil extracting. The pores within the filter were controllable by adjusting sintering conditions and properties of the polymer powder. The low oil permeability of the SLS 3D-printed filter was suitable for hydro seal.

### 3.2. Electrical and Electronic Application

Fabricating components that support electronic or sensing applications is another area where the powder-based 3D-printing technology plays a part [200].

Metal PBBJ was used to create fractal monopole antennas [201]. These complex structures, which could only be fabricated by 3D printing, reduced the material cost and improved the antenna performance with different matching and radiation patterns. The smallest feature on the antenna structure is approximately 2 mm. The 3D-printed antennas were used for 2G Hz Bluetooth and wireless local area network (WLAN) band. Another improved version using inverse fractal shape also supported 5.5 GHz WLAN frequency. The antenna consisted of various complex mesoscale structures. The complex 3D antenna design resulted in an improved antenna matching for a higher frequency region within the bandwidths compared to the conventional Sierpiński tetrahedron structure and a reduction in volume by 75% which was significant for material cost saving. To fabricate more precise features for integrated electronics, there was a need to develop powder-based 3D-printing techniques with high-resolution and better surface finishing [201]. An SLM 3D-printed Cu-15Sn waveguide was used in millimeter-wave and terahertz applications. The cross-section of the waveguide has a dimension of 3.03 × 1.55 mm at E-band, 1.73 × 1.55 mm at D-band and 0.88 × 0.45 mm at H-band. The attenuation of the waveguide was comparable to commercial nonmetallic waveguide at E-band [202].

**Figure 9:**
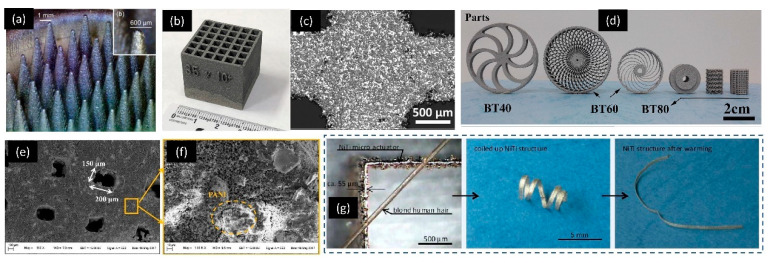
Example of various powder-based 3D-printed electrical and electronic application devices. (**a**) SS 316L monolithic corona ionizer arrays, diameter tip 300 μm. Reproduced with permission from [203]; (**b**,**c**) PBBJ 3D-printed B4C–Al composites complex collimator, minimum mesh size 1.5 mm. Reproduced with permission from [96]; (**d**) SLS 3D-printed PA11/BaTiO3 nanocomposite various complex structures. Reproduced with permission from [205]; (**e**,**f**) SEM image of SLM 3D-printed multiscale supercapacitor, pore size 150–200 μm. Reproduced with permission from [209]; (**g**) SLM 3D-printed shape-memory micro-actuators, thickness 55 µm. Reproduced with permission from [210].

Figure 9 shows some examples of 3D-printed electrical and electronic devices. Figure 9a shows an example of PBBJ 3D-printed SS 316L monolithic multi-emitter corona ionizer array with a minimum feature (tip diameter) size of 300 μm [52,203]. Another example of PBBJ 3D-printed device is shown in Figure 9b,c. This device is a complex collimator made of B4C–Al composites with a minimum mesh size of 1.5 mm [96]. PBBJ 3D-printing technique was used to create a ceramic-based device that showed piezoelectric response [204]. The minimum feature size of the device was approximately 1–2 mm. The dielectric constant of sample tested in the direction normal to the printing layers was higher than the that tested in the direction parallel to the printing layers. The device achieved 80% of the theoretical piezoelectric properties using ceramic BaTiO_3_ with only 36.77% density of conventional devices. This device showed potential for efficient and cost-effective sensing and energy harvesting.

Figure 9d shows various piezoelectric polymer/nanocomposite (PA11/BaTiO_3_)-based complex micro and mesoscale structures fabricated by SLS 3D-printing process [205]. SLS 3D-printing technique helped to create flexible and electrically conductive TPU/graphene (TPU–GE) cellular structures [206]. The smallest 3D-printed feature in the specimen was approximately 2 mm. The SLS 3D printing was especially useful for creating complex periodic structures. The 3D-printed specimen demonstrated the ability to conduct electricity due to the graphene nanoplatelets assembled on the surface of the TPU powder. The 3D-printed porous structures showed excellent strain sensitivity in negative piezoresistive behavior. The TPU–GE porous structures showed potential applications in wearable sensors, soft implants and dielectric elastomer actuators. SLS 3D printing also proved itself to be a suitable candidate for creating 3D-molded interconnect devices (MID) [207,208]. SLS 3D printing could selectively metallize the surface of PA12 catered for rapid prototyping and small-scale production of 3D-MIDs. The metallization of the PA12 surface increased the mechanical properties and heat conductivity of the 3D-printed PA12 components. This was achieved via coating the SLS-polymer with special paint containing additives for laser direct structuring followed by copper deposition in selected regions. The smallest feature of the interconnects demonstrated was approximate 1–2 mm.

Multiscale supercapacitor based on Fe–Ni alloy has been fabricated using SLM 3D-printing process [209]. This multiscale supercapacitor has well-arranged porous structure of a minimum feature (pore) size of 150–200 μm (Figure 9e,f). These porous structures increase the specific surface area of the 3D-printed multiscale supercapacitor, which leads to a high specific capacitance of the 3D-printed device. Micro-actuators have been fabricated using SLM 3D-printing process with a minimum feature (width) of around 50 μm without compromising the properties of shape-memory alloys (Ni-Ti) [210]. Figure 9g shows the Ni–Ti micro actuator phase transformation under elevated temperature.

Another area where powder-based 3D printing could contribute is the manufacturing of electrodes. The aim is to fabricate pure copper electrodes with the highest possible density for lowest possible electrical resistivity using minimal laser power [211]. The minimum feature size of a thin electrode was approximately 200 µm. Heat treatment reduced the porosity of the 3D-printed copper electrode. Consequently, a pure copper electrode achieved a lower electrical resistivity compared to an electrode made of other 3D-printing material such as aluminum alloy. Although 3D-printed electrodes have not yet achieved the resistivity of the conventional copper electrodes, it is low enough to meet the requirements of certain applications. The advantages of 3D-printed electrode include flexibility for custom design and higher slot fill factor with high power density, which could reduce the size and weight of the electrical motor. Electrode with larger slot fill factor increases the optimum power that the electrode can transfer. There was also some effort in using SLM to 3D-print electrode with a complex shape design that required long process time for conventional method [212]. The 3D-printed electrode functioned well as pseudo capacitor, catalytic setup for making oxygen and pH sensor. The performance of the 3D-printed electrode is similar to commercial electrode. The overall dimension of the electrode was in the range of centimeters with mesoscale circular structure as narrow as approximately 0.4 mm. PBBJ 3D-printing process was used to make 1 mm thick graphene-based electrode on top of the porous graphene surface to provide a large surface area [213]. The fabricated graphene electrode was used in a supercapacitor. A similar technique was used to fabricate graphene hydroxyapatite nanocomposite structures. The graphene improved the flowability of the nanocomposite material for better powder spreadability during printing. The compressive strength of the 3D-printed cylindrical parts with 4 mm diameter was excellent, and those parts were suitable for load-bearing bio-applications. The nanoparticles in agglomerated graphene oxide sheet enhanced the mechanical strength of the 3D-printed cylinder [214]. Powder bed 3D printing offers a flexible technique for the fabrication of personalized electronics. One of the smallest functional devices contained steel electrodes 3D printed using SLM. The overall dimension of the electrode was a rectangle of 6 × 9 centimeters with a smallest feature size of 0.1–0.2 mm in the vertical direction which had reached the limit of powder-based 3D printing because each powder layer was approx. 100 µm [215].

### 3.3. Industrial, Mechanical and Aerospace Applications

Powder-based 3D printing helps to tackle other challenges by creating multilayer 3D structures. The automotive, mechanical and aerospace industries are promising fields where powder-based 3D printing is readily adopted [37,79,120,138,216,217,218]. There are numerous examples of powder-based 3D-printed micro and mesoscale components or devices that have been used in these industries, including functional parts, functional prototypes, tooling, lightweight components, repair of part, heat transfer devices, mechanical and thermal switches and fuel nozzles among others [37,79,128,137,139,217,218,219,220,221,222,223,224].

**Figure 10:**
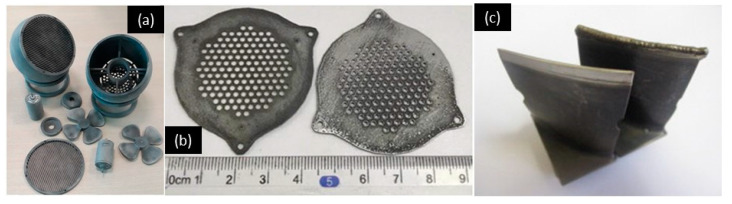
Example of various powder-based 3D-printed mechanical and aerospace application devices (**a**) MJF 3D-printed functional part, feature size 2 mm. Reproduced with permission from [109]; (**b**) SLM 3D-printed ion optics grids. Reproduced with permission from [226]; (**c**) repair of compressor blade by PDMD 3D-prining process, minimum feature size 0.6409 mm to 1.2218 mm. Reproduced with permission from [222].

Figure 10a shows MJF 3D-printed functional parts. This 3D-printed fan has a small feature size of 2 mm [128]. SLS 3D-printing technique was used to create honeycomb and reentrant core structures for motorsport and aerospace applications [224]. The materials used to make these structures were carbon fiber, aluminum alloy and PA 12. Honeycomb structures with features of 0.42 mm was 3D-printed and tested for its strength and stiffness. The 3D-printed reentrant cores exhibited greater stiffness to weight ratio by taking advantage of 3D printing to reduce the cell wall thickness. The SLM 3D-printing process can print mesoscale glass structures with different features of approximately 0.5 mm. This is a leap forward from conventional glass forming method by offer a higher degree of flexibility in designing complex 3D structures [123]. In the area of acoustic sound damping, SLS 3D printing was used to create multilayer micro-perforated panels (MPPs) for sound absorption with a tunable wideband [225]. The SLS 3D printing was able to produce different air gap distances, inter-layer distances and geometries which resulted in a wider frequency bandwidth with a higher overall absorption coefficient and tunable adsorption peaks at different frequencies compared to the traditional single layer MPPs. The smallest feature within the panel was 0.9 mm.

3D printing has been used to manufacture aerospace parts with high-performance and long-service-life ion engine grid [226]. The engine grids with the smallest feature of about 1 mm was 3D-printed by SLM accurately with titanium and molybdenum with an excellent surface finish, which outperformed conventional optics (Figure 10b). This optical component was used as accelerator ion optics in the Helicon ion thruster system [226]. Functionally graded materials (FGM) with a gradual transition from one material to another were realized with SLM 3D printing [227]. This was achieved by incorporating multiple selective powder delivery arrays into a SLM system. This system allowed a maximum of six different materials to be deposited point-by-point onto the powder bed. The turbine disk fabricated by the 3D FGM had a thickness of approximately 1 mm in the blade. The flexibility of multiple material deposition methods in SLM empowers the creation of FGM components. Figure 10c shows an example of a compressor blade repairing via PDMD 3D-printing process with a minimum feature (leading edge) size of 0.6409 mm.

**Figure 11:**
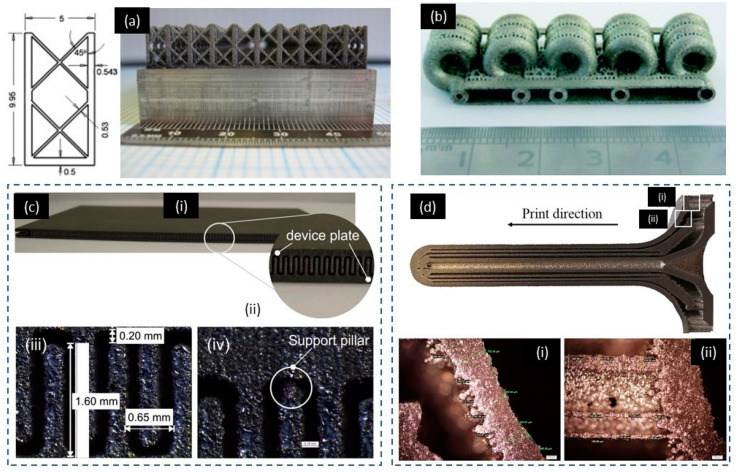
Example of Powder-based 3D-printed heat transfer devices and heat switch (**a**) SLM 3D-printed heat transfer device. Reproduced with permission from [229]; (**b**) SLM 3D-printed mesoscale flow reactors. Reproduced with permission from [231]; (**c**) SLM 3D-printed compact heat switch. Reproduced with permission from [121]; (**d**) high-temperature aerospace resistojet heat exchanger 3D-printed by SLM 3D-printing process. Reproduced with permission from [233].

The heat transfer device was another area SLM 3D printing could improve. SLM 3D printing is able to create any arbitrary shapes with heat transfer capabilities better than the ones fabricated by conventional machining methods [228]. SLM 3D printing enables innovative design of complex shapes, internal cooling channels and porous structures, which makes it an excellent choice for making heat transfer devices. The smallest feature 3D-printed on these devices was approximately 0.5 mm. Five types of heat sink structures were designed, including cylindrical, rectangular, rounded corned rectangular fin array, elliptical array and lattice [229]. SLM 3D printing successfully manufactured all five types of heat sinks. This study showed that SLM 3D-printing process improved the efficiency of the heat sink. Figure 11a shows an example of SLM 3D-printed lattice heat sinks with a minimum feature size of 0.53 mm [229]. Similarly, the cooling performance of heat fins of various shapes was evaluated [230]. In this study, the minimum feature size of the 3D-printed fin structure varied from 300 µm to 1260 µm. Effective cooling was achieved by using these improved structures with high-thermal flux. The capability of the SLM 3D-printer to produce flow reactor with internal flow channel was demonstrated. The diameter of the internal channels was in the range of 1 mm to 2 mm (Figure 11b) [231]. The residual powders were successfully removed from all channels, indicating that SLM had the capacity to produce mesoscale hollow internal structures. The 3D-printed compact heat switch that operated at cryogenic temperature was successfully manufactured [232]. This SLM 3D-printed switch was a compact flat-panel gas–gap heat type with a minimum feature size of 200 μm–500 μm (Figure 11c). Figure 11d is another example of SLM 3D-printed high-temperature aerospace resistojet heat exchanger with varying wall thickness ranging from 660 μm to 800 μm. However, the minimum wall thickness of supporting structures was measured to be 100 μm [233].

## 4. Future Development of Powder-Based 3D Printing

Demand for powder-based 3D-printing technologies will escalate in the future due to their many advantages including the capability to make customized microscale and mesoscale devices that can be tailored for specific applications.

High surface roughness and porosity due to limited printing resolution represent one of the constraints to use powder-based 3D-printed parts as microfluidic devices. Despite the limited 3D-printing resolution, many applications that are not sensitive to 3D-printing resolution can be realized. Powder-based 3D printing is inherently more porous and rougher compared to liquid resin-based 3D printing. Although newer powder-based processes, such as MJF, could produce parts with low porosity, they still heavily rely on post-printing processing to reduce the surface roughness. Powder-based 3D printing is prone to subsurface depletion zone and pores which may cause unevenness on the surface. The unevenness and high surface roughness can cause fluid leakage in 3D-printed fluidic devices, such as microfluidic chips, due to the weak bonding between the microfluidic chip and substrate for sealing. However, some of these shortcomings can be addressed with mechanical polishing or surface coating. Optical transparency is a desired feature of 3D-printed microfluidic chips. It allows user to observe the behaviors and fluids and objects through the chip. Non-powder-based 3D-printing technology using photopolymerization is comparatively more suitable for microfluidic devices as it can print material with excellent optical transparency. With this capability, SLA has been used to develop microfluidic or biosensing applications with transparent windows or channels to observe the physical phenomenon within the microchannel [164,234]. The SLA 3D-printing technique suffers from slow production time, limited build size and problems associated with the high viscosity of the resin, but it is capable of printing transparent chip with a resolution higher than powder-based 3D-printing techniques [235]. Furthermore, SLA 3D printing is able to directly pattern cells, cell matrix and other functional molecules such as growth factors for potential fabrication of tissue and organ construct [236,237,238]. The powder-based 3D printing avoids shortcomings of SLA and other 3D-printing techniques based on photopolymerization, but it suffers from limited resolution and undesired optical properties (e.g., non-transparent). Fortunately, a new-generation of powder-based 3D-printer attempts to address some of these challenges [48,51,58,59,60,123,239]. Once these barriers are lifted, powder-based 3D printing will be more widely applicable.

MJF is a candidate to produce high-resolution microfluidic channel down to 250 µm. A range of biocompatible materials are available for MJF. Currently MJF does not offer transparent base materials. However, there are other ways to create transparent lids for microfluidic chips printed by MJF, such as bonding a glass coverslip to the device. In addition, a voxel transforming agent is under way. The transforming agent could change the color, surface properties, elasticity, strength, electrical conductivity and translucency of each building block of MJF parts. This development can address some of the limitations in the future. In addition, the voxel transforming agent will offer additional flexibility in creating customizable complex functional parts within a single print job.

In combination with artificial intelligent and other industry 4.0 technologies, powder-based 3D-printing technology will enable a new-generation of personalized products that are customizable on-demand. By designing and printing parts according to customer’s requirements on-demand, powder-based 3D printing reduces the need for businesses to hold large inventories by offering rapid production with a high flexibility and a high degree of customization.

## 5. Conclusions

The capability of powder-based 3D-printers to print micro and mesoscale features has empowered a broad range of applications with significant improvements compared to conventional manufacturing methods. These improvements include the flexibility in designing complex structure, usage of different materials within a single design, reduced material cost for manufacturing and on-demand manufacturing of customized products. Despite all the advances in powder-based technology, there are still major gaps in this field, including higher material cost, longer printing time, laborious post-processing, thermal distortion that leads to warping and limited material selection. Nevertheless, with continuous improvements on the dimensional accuracy, printing resolution and production speed, when combined with all the advantages of powder-based 3D printing, powder-based 3D printing will overcome these limitations and propel its wide adoption for industrial scale production of microscale and mesoscale features in the future.

## Figures and Tables

**Table 1:** Different powder-based 3D-printing process capabilities and applications.

3D-Printing Process	Layer Thickness (μm)	Minimum Feature (μm)	Main Applications	References
Powder bed binder jetting (PBBJ)–ceramic, composites	2–300	22–500	Mold manufacturing, microporous bioceramic implants, bioresorbable devices, Surgical templates, drug delivery system, Implant with various medicines, highly porous tablet, orodispersible dosage forms, extended-release tablet	[71,91,99,145,146,147,148,149]
PBBJ–polymer and metal	20–100	100–500	Lattice structures, mold manufacturing, prototyping, implants	[91,99,145,146,147,150]
Multijet fusion (MJF)	80–100	250–500	Lattice structures, prosthetics, functional part, dental aligners, orthotics, robotic arm/grip, motorbike manifold, fluid management systems	[87,106,108,109,151]
Selective laser sintering (SLS)	76–100	40–100	Various types of non-porous and porous structures, scaffolds, biodegradable scaffolds, biomedical fabrication, dental components, craniofacial and joint implants, modified-release and immediate-release tablets, orally dissolving tablet	[99,126,146,147,148,149,150,152,153,154,155]
Selective laser melting (SLM)	20–100	40–200	Electronics, aerospace, scaffolds, biodegradable scaffolds, biomedical fabrication, cervical, vertebral body replacement, porous dental implants, heat exchanger, cryogenic switch, heat sinks	[3,33,146,152,156]
Electron beam melting (EBM)	50–200	100–200	Various types of non-porous and porous structures, scaffolds, turbine blade manufacturing and repair	[3,33,152]
Powder directed energy deposition (PDED)	200–800	500–3000	Repair of bespoke parts, biomedical fabrication, knee and hip implants, turbine blade manufacturing and repair	[3,33,152]

**Table 2:** Summary of all powder-based devices with miniaturized features.

Powder-Based Miniaturized Device	Smallest Feature Size	3D-Printing Technique	Material	Short Description	References
Near zero-order release dosage forms (biomedical application)	2.8 mm	PBBJ	Kollidon SR and hydroxypropylmethyl cellulose	PBBJ 3D-printed water-soluble compound enabled a controlled drug released rate based on different ratio of the two polymers	[189]
Calcium phosphate powder-binder system for patient-specific implants (biomedical application)	1 mm	PBBJ	Tetracalcium phosphate, β-tricalcium phosphate and calcium sulfate dihydrate	Ceramic bone substitute and scaffold for bone tissue engineering are tested with in vitro cytocompatibility testing	[157]
Drug delivery devices (biomedical application)	1 mm	Customized PBBJ	Paracetamol, lactose, PVP K30, mannitol and colloidal silicon dioxide	Oval fast-disintegrating tablet for drug release is 3D-printed with accelerated drug releasing profile	[186]
3D-printed fast-disintegrating tablet (biomedical application)	1.4 mm	Customized PBBJ	Acetaminophen, methylene blue, colloidal silicon dioxide and polyvinylpyrrolidone	A fast-disintegrating tablet achieved fast dissolving properties	[190]
3D-printed scaffolds with minimum (biomedical application)	330 μm–1 mm	PBBJ	Stainless steel 316	Various sizes, shapes and lattice structure designs are 3D-printed, evaluated process parameters, dimensional and mechanical properties	[171]
3D-printed patient-specific dental implants. (biomedical application)	0.5 mm	PBBJ	Nickel-based alloy 625	Patient-specific complex metal partial denture framework	[181]
3D-printed complex collimator device (electrical and electronic application)	1.5 mm	PBBJ	B4C–Al composites	This highly dense complex collimator is found to be good for neutron scattering	[96]
Thick graphene-based electrodes (electrical and electronic application)	~1 mm	PBBJ	Exfoliated graphene oxide powder	Porous graphene-based high-performance supercapacitor is 3D-printed with PBBJ	[213]
Graphene hydroxyapatite nanocomposite structures (electrical and electronic application)	4 mm	PBBJ	Graphene oxide, hydroxyapatite nanocomposite	Graphene/HAP nanocomposite 3D-printed cylinder with 125 μm layer thickness proved to have excellent compressive strength	[214]
3D electronic applications (electrical and electronic application)	~1 mm	PBBJ	Gold, silver and copper	Conductive paths and other electronic components are 3D-printed for seamless integration with other electrical and electronic functionality	[200]
3D printing of fractal antennas (electrical and electronic application)	~2 mm	Metal PBBJ	Stainless steel	The complex inverse Sierpiński tetrahedron fractal antenna proved functional at two WLAN bands with 23% less material used	[201]
3D-printed monolithic multi-emitter corona ionizer (electrical and electronic application)	300 μm	PBBJ	SS 316L	Demonstrated the design, manufacture and characterization methods for 3D-printed corona ionizer	[52,203]
3D-printed induced orthotropic functional ceramic (electrical and electronic application)	~1–2 mm	PBBJ	Barium titanate	Ceramic-based device for generating piezoelectric response	[204]
3D-printed patient-specific ankle-foot orthoses (AFO) (biomedical application)	1.2 mm	MJF	PA12	The 3D-printed AFO significantly improved the speed and stride length of the stroke patients	[108]
3D-printed functional part. (industrial, mechanical applications)	2 mm	MJF	PA12	Demonstrated the capability of MJF, to printed functional parts with high accuracy	[128]
3D-printed scaffold (biomedical application)	40–400 μm	SLS	Polycaprolactone	Effective for cell attachments	[126,172]
3D-printed porous Ti–6Al–4V scaffold (biomedical application)	723 μm	DMLS	Ti–6Al–4V	Bone defect repair example of porous Ti–6Al–4V scaffold	[177]
3D-printed scaffold (biomedical application	0.5–1.2 μm	SLS	Ceramic-based material,	Bioactivity improvement, better properties	[173,174].
3D-printed orally disintegrating printlets (biomedical application)	2 mm	SLS	Hydroxypropyl methylcellulose and vinylpyrrolidonevinyl acetate copolymer powders	Orally disintegrating tablet with tunable drug release profile	[192]
3D-printed macrocapsule for cell-based therapies (biomedical application)	0.5 mm–1 mm	SLS	Alginate-poly-L-lysine	Microcapsule which can produce therapeutic proteins	[191]
3D-printed electronic circuit carriers (electrical and electronic application)	~1 mm	SLS	Copper powder	Selectively metallize PA12 surface to form electrical interconnects	[207,208]
3D-printed thermoplastic polyurethane/graphene cellular structure (electrical and electronic application)	~2 mm	SLS	Graphene and thermoplastic polyurethane	Porous structure which is both electrically conductive and flexible	[206]
3D-printed filter (chemical industry applications)	1.5 mm	SLS	MOF copper (II) benzene-1,3,5-tricarboxylate	SLS 3D-printed filters that can filter out precious metal from liquid	[198]
3D-printed sandwich material for motorsport applications (aerospace devices)	~1 mm	SLS	PA12	SLS 3D-printed core structures rival the performance of common aluminum honeycomb sandwich material in term of strength and stiffness	[224]
SLS 3D-printed filter for gas separation (chemical industry applications)	~2 mm	SLS	Brass and polycarbonate/nickel and polyamide/brass, solder and colophony/nickel, solder and colophony	SLS 3D-printed filter for separation of concomitant gases	[199]
Multi-perforated panels (industrial, and mechanical application)	0.9 mm	SLS	Polyamide 12	SLS 3D-printed panel for sound damping	[225].
AM assisted manufacturing of bipolar plate in fuel cells (electrical and electronic application)	1 mm	SLS, SLM	Fusion of titanium and gold, stainless steel	3D-printed metal flow field plate gives comparable performance in mass transport compared to conventional machining process	[193]
3D-printed complex implant structures (biomedical application)	200 μm	SLM	Zn	3D-printed, biodegradable Zn based metals cardiovascular stents	[178]
3D-printed implant (biomedical application)	~0.26 mm	SLM	Ti–6Al–4V	Biocompatible implant with porous structure for tissue regeneration	[175]
3D-printed implant for lower jaw (biomedical application)	~1 mm	SLM	Titanium	Customized implant	[176]
3D-printed micro-bore columns for reversed-phase liquid chromatography (biomedical application)	0.9 mm	SLM	Ti–6Al–4V powder	3D-printed chromatographic column for separation of proteins and peptides	[194]
Rectangular waveguide for millimeter-wave application (electrical and electronic application)	0.43 mm	SLM	Cu-15Sn	A mechanically robust waveguide for D, E and F band without post electroplating and assembling	[202]
Metal electrodes for electrochemical devices (electrical and electronic application)	~1 mm	SLM	Stainless steel (316L)	3D-printed electrodes as pseudo capacitor, oxygen evolution catalyst and pH sensor	[215]
3D-printed metal electrodes (electrical and electronic application)	~0.4 mm	SLM	Stainless steel	Helical stainless steel electrodes had been coated with IrO2 for pH sensor application	[212]
3D-printed multiscale supercapacitor (electrical and electronic application)	150–200 μm	SLM	Fe–Ni alloy	Well-arranged porous structure increases the specific surface area, which leads to a high specific capacitance of device	[209]
3D-printed pure copper made for electromagnetic applications (electrical and electronic application)	200 μm	LPBF	Copper	Electrical coil with various shapes and hollow centers is made and testing shows its potential to be used in electric motors, antenna and electromagnetic applications	[211]
Ion optics for electric propulsion (aerospace devices)	~1 mm	SLM	Molybdenum, combinations of molybdenum and titanium	3D-printed grids with sputtering erosion patterns are made and tested as electric propulsion parts	[226]
3D-printed FGM turbine disk (aerospace devices)	~1 mm	SLM	Spherical 316L stainless steel and Cu10Sn copper alloy	SLM fabricated 316L/Cu10Sn turbine that has higher hardness than conventional processes	[227]
SLM 3D-printed heat transfer devices (devices for other applications)	~0.5 mm	SLM	Stainless steel, aluminum, Ti–6Al–4V, steel–nickel, Titanium, etc.	Customized 3D-printed heat transfer device for cooling applications	[228]
3D-printed various lattice heat sinks device (aerospace devices)	0.53 mm	SLM	Aluminum 6061	3D-printing process improved the efficiency of the heat sink.	[229]
3D-printed various fin structures (aerospace devices)	300 µm–1260 µm	SLM	Aluminum alloy (AlSi10Mg)	These 3D-printed fin structures are can be utilized in devices for efficient cooling	[230]
3D-printer mesoscale flow reactors (aerospace devices)	1 mm–2 mm	SLM	Stainless steel	Internal flow channel was demonstrated.	[231]
3D-printed compact heat switch (aerospace devices)	200 μm–500 μm	SLM	Ti–6Al–4V	Mesoscale hollow internal structures, operates at cryogenic temperature	[232]
3D-printed high-temperature aerospace resistojet heat exchanger (aerospace devices)	200 μm–800 μm	SLM	Stainless steel	Design, manufacture and characterization of a high-temperature resistojet for all-electric spacecraft	[233]
Manufacturing of glass with various shapes with micro/macro scale resolution (Biomedical, chemical, industrial, and mechanical applications)	~0.5 mm	LPBF	Soda lime silica glass	High level of complexity of small-scale glass structures is 3D-printed opening possibilities for applications in chemistry, biomedical and decorative glass industries	[123]
Metallic implants based on laser and electron beam powder-based AM (biomedical application)	~0.3 mm	SLM, EBM	316L stainless steel, titanium-6aluminum–4vanadium and cobalt–chromium	EBM and SLM 3D printing enable mass customized implant at lower cost compared to conventional molding technique	[27]
Marine species tracking tag (biomedical application)	1 mm	EBM	Titanium	A sharp tag with textured surface for easy penetration of marine species‘ skin for tracking purpose	[166]
3D-printed disc biocompatibility test (biomedical application)	2 mm	EBM	Ti–6Al–4V powder	Biocompatible disc for fibroblast cell culture	[182]
3D-printed mesh for intercellular cell communication and osteoincorporation (biomedical application)	~1 mm	EBM	Ti–6Al–4V powder	Biocompatible mesh for growth of mouse preosteoblast MC3T3-E1 subclone 4 cell line	[179]
3D-printed anodized mesh structure (biomedical application)	~0.5 mm	EBM	Ti–6Al–4V powder	Biocompatible mesh for growth of mouse preosteoblast MC3T3-E1 subclone 4 cell line	[183]
3D-printed scaffold for cell culture (biomedical application)	0.7 mm	EBM	Ti–6Al–4V powder	Biocompatible foamed structure for growth of mouse preosteoblast MC3T3-E1 subclone 4 cell line	[184]
3D-printed scaffold for titanium implant (biomedical application)	0.7 mm	EBM	Ti–6Al–4V powder	Biocompatible scaffold for osseointegration and angiogenesis testing	[185]
3D-printed rough and porous dental implants (biomedical application)	500 µm	EBM	Ti–6Al–4V	Dental implants facilities bone ingrowth and strengthens bone bonding	[180]
Repair of compressor blade (aerospace devices)	0.6409	PDMD	Inconel 718	Compressor blade repairing using PDMD 3D-printing process	[222]
